# Association between pet ownership and sleep in the Swedish CArdioPulmonary bioImage Study (SCAPIS)

**DOI:** 10.1038/s41598-021-87080-7

**Published:** 2021-04-02

**Authors:** Lieve T. van Egmond, Olga E. Titova, Eva Lindberg, Tove Fall, Christian Benedict

**Affiliations:** 1 Department of Neuroscience, Uppsala University, Husargatan 3, Box 593, 751 24 Uppsala, Sweden; 2 Department of Surgical Sciences, Unit of Medical Epidemiology, Uppsala University, Uppsala, Sweden; 3 Department of Medical Sciences, Respiratory, Allergy and Sleep Research, Uppsala University, Uppsala, Sweden; 4 Department of Medical Sciences, Molecular Epidemiology and Science for Life Laboratory, Uppsala University, Uppsala, Sweden

**Keywords:** Risk factors, Sleep

## Abstract

Preliminary findings suggest that pets may impact the owner’s sleep. By using data from the Swedish CArdioPulmonary bIoimage Study (SCAPIS) cohort, we aimed to investigate the association of pet ownership with the following self-reported sleep outcomes in 3788 to 4574 participants: (i) achieving the recommended daily sleep duration for adults (i.e., at least 7 h per day); (ii) sleep quality as measured by the Pittsburgh Sleep Quality Index (a score of > 5 indicating poor sleep quality); and (iii) difficulty falling or staying asleep. Sleep metrics were not associated with pet ownership, dog ownership, and dog walking when controlling the logistic regression for possible confounders (e.g., shift work, lack of social interaction, and chronic stress). In contrast, cat ownership was associated with a higher odds ratio of failing to achieve the recommended duration of 7 h of sleep per day (adjusted odds ratio [95% CI]:1.18 [1.02, 1.37] versus non-cat owners). Our findings suggest that certain pet groups might have a more significant impact on the owner’s sleep than others. As the observed association between cat ownership and short sleep duration might be a chance finding, this observation should be seen as hypothesis-generating only.

## Introduction

The potential therapeutic value of pets to improve sleep has been the subject of recent research. The most extensive study to date, a cross-sectional examination of 6,575 participants of the Whitehall II study aged between 59 and 79, revealed that pet owners had less trouble falling asleep than non-pet owners^1^. Companionship, security, physical activity (e.g., due to dog walking), and relaxation may represent possible mechanisms through which pets facilitate the owners’ ability to fall asleep. On the other hand, in the same cohort, pet ownership was also associated with feeling more tired after waking up^1^, possibly because pet owners may be waking due to their pets. The results of smaller studies painted a similarly complex picture of the association between pet ownership and sleep. A questionnaire study involving 801 subjects (ages ranged from 20 to 50 years) found that men who owned a pet reported better subjective sleep quality^2^. Using accelerometry to measure sleep objectively for seven nights, a separate study involving 40 healthy dog owners without sleep disorders (mean age 44 years) found that sleep efficiency was lower if the dog was on the bed instead of merely in the room^3^. Extending this finding, another study involving 150 patients (age range not specified) found that about 20% of the pet owners described their pets as disruptive^4^. The heterogeneity of the results and the fact that this topic is not well-researched highlights that more studies are required.

In the present study, using survey data from the Swedish CArdioPulmonary bioImage Study (SCAPIS), we aimed to examine the following hypotheses: (i) pet owners have higher odds of achieving the recommended daily sleep duration for adults (i.e., at least 7 h per day); (ii) pet owners report better sleep quality as measured by the Pittsburgh Sleep Quality Index (PSQI; see ref.^5^); and (iii) pet owners have fewer complaints of difficulty falling or staying asleep when compared to non-pet owners. We also investigated if these possible associations exist among dog or cat owners.

As regular physical activity has been shown to improve various sleep parameters, e.g., sleep onset latency and sleep quality^6–8^, we also investigated whether dog owners who reported to be mainly responsible for dog walking would exhibit better sleep.

## Material and methods

### Population and study design

The SCAPIS study is a Swedish nationwide population-based cohort mainly designed to research cardiovascular and chronic obstructive pulmonary diseases. Between 2013 and 2018, 30,154 men and women aged 50 to 65 were recruited from a random population sample by six Swedish university hospitals (Gothenburg, Linköping, Malmö/Lund, Stockholm, Umeå, and Uppsala). The primary aim of SCAPIS is to improve the risk prediction of cardiopulmonary diseases and optimize the ability to study disease mechanisms^9^.

**Figure 1:**
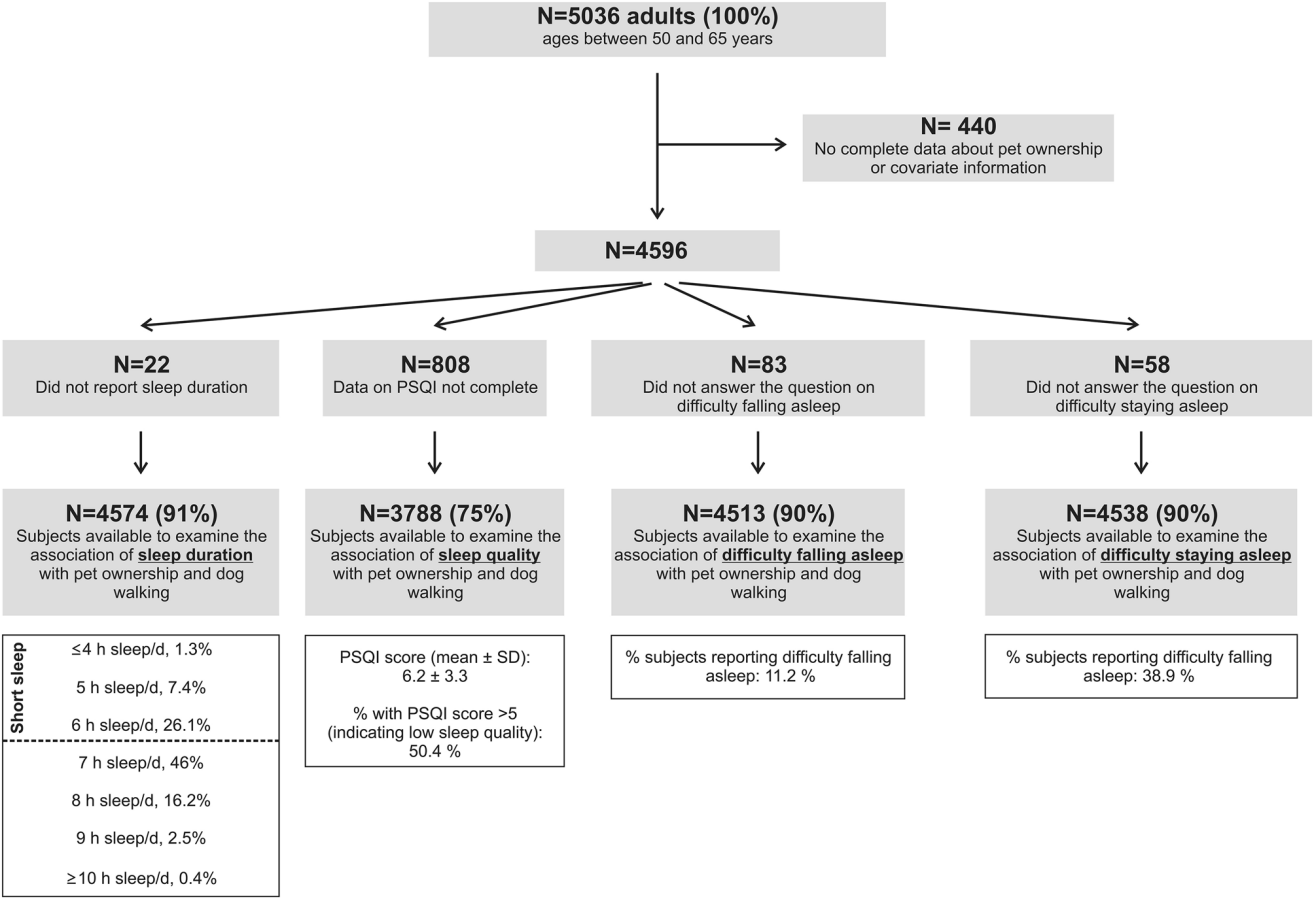
Flowchart.

In addition to surveying the general health status and lifestyle habits, all Uppsala participants (but not the other areas) were also asked about pet ownership (see below). Following exclusions (detailed in Fig. 1), data from 4,574 subjects from the Uppsala cohort were available to examine the association between pet ownership and sleep duration, 3788 subjects for the sleep quality analysis, 4513 subjects for the difficulty falling asleep analysis, and 4538 subjects for the difficulty staying asleep analysis, respectively. The Regional Ethical Review Board in Uppsala (*Etikprövningsmyndigheten Uppsala; approval number DNR2019-05343*) approved the study, and all participants provided written informed consent. All investigations were conducted in accordance with the Declaration of Helsinki.

### Exposure—pet ownership

Pet ownership was assessed by the question: *“Do you have a pet in the household?”*. Participants could answer with *“yes”*, *“no”*, or *“I can’t or don’t want to answer”*. The answer *“I can’t or don’t want to answer”* was treated as missing data. If answering yes to the pet ownership question, participants could fill in which and how many pets they had, divided into *“dog”*, *“cat”*, *“rabbit”*, *“bird”*, *or “other”*. Dog owners were also asked about their dog walking engagement. Possible answer options were: “*I mainly take care of the dog’s daily exercise*”, *“I take care of about half of the dog's daily exercise”, “I take care of a small part of the dog's daily exercise”, “I have no dog”, and “I can’t or don’t want to answer”.* Dog owners who chose the answer options *“I have no dog”* or *“I can’t or don’t want to answer”* were treated as non-dog owners in the analysis regarding the association between dog walking and sleep.

### Outcome—sleep

Participants were asked, *“How many hours do you normally sleep during a day”* with the following answer options: *“4 h or less per day”*; *“5 h per day”*; *“6 h per day”*; *“7 h per day”*; *“8 h per day”*; *“9 h per day”*; and *“10 or more hours per day”.* In the present analysis, *“7 h*” or more were classified as achieving the recommended daily sleep duration. Note that this question was part of the SCAPIS core questionnaire, i.e., it did not derive from the PSQI where the respondent is typically asked several questions regarding sleep, including a question on how many hours (s)he habitually slept during the past month.

Participants’ sleep quality was measured using the PSQI^5^. As described elsewhere, the PSQI surveys various sleep characteristics during the past month, such as sleep latency (i.e., how long it takes to fall asleep). Each item is scored on a 0–3 interval scale (0 = not during the past month; 1 = Less than once a week; 2 = once or twice a week; and 3 = Three or more times a week). The answer *“Three or more times a week”* to these questions was classified as having this sleep problem. Overall, a score of > 5 defines poor sleep quality^5^.

In SCAPIS, participants could also choose the answer option “*I can’t or don’t want to answer*” when answering the PSQI components. This answer option does typically not exist in the PSQI^5^. To ensure that our results can be compared with past and future investigations using the PSQI, participants who answered any questions of the PSQI with “*I can’t or don’t want to answer*” were excluded from the analysis.

The PSQI questions “*During the past month, how often have you had trouble sleeping because you cannot get to sleep within 30 min?*” and “*During the past month, how often have you had trouble sleeping because you wake up in the middle of the night or early morning?*” were used in a separate analysis to evaluate whether pet ownership associated with difficulty falling and difficulty staying asleep, respectively. The answer “*Three or more times a week*” to these questions was classified as having this sleep problem.

### Confounders

We applied d-separation criteria on directed acyclic graphs (DAGs; see ref.^10^) to identify potential confounders to include in the regression model (see Supplementary Figure S1). DAGs is a widely used method to depict graphically assumed causal relationships between predictor, outcome, and confounder variables.

For the analysis, education was divided into university degree vs no university degree. A high level of social interaction was defined as meeting more than two close friends every week. Physical activity status was divided into ≥ 2 h of modest to heavy exercise/week vs < 2 h of modest to heavy exercise/week. Alcohol consumption frequency was divided into ≥ 2 times/week vs < 2 times per week. The following question assessed socio-economic stability: “*Could you raise 20,000 SEK within a week if required?”* (answer options: yes, no). Furthermore, subjects reported whether they work night shifts, have been diagnosed with sleep apnea, experienced a high-stress load during the past 12 months (yes, no), and if they currently smoke. Some questions also had the response option “*I can’t or don’t want to answer*”. Hence, these confounders were entered as variables with three answer options into the analysis. Diabetes was either confirmed by questionnaire (yes, no) or when participants without previously known diabetes had a blood glucose level of at least 7 mmol/l or an HbA1c level of at least 48 mmol/mol. The weight and height were measured during the physical SCAPIS examination and used to calculate BMI. Age was recorded at the time of the examination.

### Statistical analysis

All analyses were performed using IBM SPSS Statistics 24 (SPSS Inc. Chicago, IL, USA). Logistic regression was used to investigate the association of pet ownership (any pet, dogs, or cats) and dog walking with sleep. Results from adjusted (including all confounders described above) logistic regressions are reported. Overall, a *P* value < 0.05 was considered significant.

## Results

### Cohort characteristics

As shown in Table 1, all subsamples used in the present analysis had a nearly balanced gender distribution. The vast majority of participants reported a stable socio-economic status, about half had a university degree, and slightly more than 10% stated that they were less socially interactive. For more cohort characteristics, see Table 1.

**Table 1:** Cohort characteristics.

	SCAPIS subsamples			
	Sleep duration	PSQI	DIS	DSA
Number of subjects (n)	4574	3788	4513	4538
Age (years)	57.7 ± 4.4	57.5 ± 4.4	57.7 ± 4.4	57.6 ± 4.4
BMI (kg/m^2^)	27.0 ± 4.4	26.7 ± 4.2	27.0 ± 4.4	27.0 ± 4.4
**Sex**				
Men	2207 (48.3)	1856 (49.0)	2172 (48.1)	2187 (48.2)
Women	2367 (51.7)	1932 (51.0)	2341 (51.9)	2351 (51.8)
**Nightshift work**				
No	4117 (90.0)	3456 (91.2)	4065 (90.1)	4088 (90.1)
Yes	330 (7.2)	241 (6.4)	323 (7.2)	324 (7.1)
Don’t know/don’t want to report	127 (2.8)	91 (2.4)	125 (2.8)	126 (2.8)
**Stable socio-economic status**				
No	246 (5.4)	157 (4.1)	242 (5.4)	248 (5.5)
Yes	4245 (92.8)	3591 (94.8)	4190 (92.8)	4211 (92.8)
Don’t know/don’t want to report	83 (1.8)	40 (1.1)	81 (1.8)	79 (1.7)
**Sleep apnea diagnosis**				
No	4368 (95.5)	3636 (96.0)	4315 (95.6)	4333 (95.5)
Yes	177 (3.9)	137 (3.6)	173 (3.8)	176 (3.9)
Don’t know/don’t want to report	29 (0.6)	15 (0.4)	25 (0.6)	29 (0.6)
**Diabetes diagnosis**				
No	4201 (91.8)	3506 (92.6)	4149 (91.9)	4170 (91.9)
Yes	373 (8.2)	282 (7.4)	364 (8.1)	368 (8.1)
**Current smoker**				
No	4109 (89.8)	3440 (90.8)	4059 (89.9)	4077 (89.8)
Yes	405 (8.9)	301 (7.9)	395 (8.8)	401 (8.8)
Don’t know/don’t want to report	60 (1.3)	47 (1.2)	59 (1.3)	60 (1.3)
**Perceived stress level in the past year**				
Low	3580 (78.3)	3021 (79.8)	3532 (78.3)	3549 (78.2)
High	964 (21.1)	760 (20.1)	950 (21.1)	957 (21.1)
Don’t know/don’t want to report	30 (0.7)	7 (0.2)	31 (0.7)	32 (0.7)
**Social interaction**				
Low	591 (12.9)	432 (11.4)	585 (13.0)	588 (13.0)
High (> 2 people/week)	3949 (86.3)	3338 (88.1)	3898 (86.4)	3918 (86.3)
Don’t know/don’t want to report	34 (0.7)	18 (0.5)	30 (0.7)	32 (0.7)
**Alcohol consumption frequency**				
Low	2882 (63.0)	2306 (60.9)	2837 (62.9)	2855 (62.9)
High (≥ 2 times/week)	1677 (36.7)	1472 (38.9)	1661 (36.8)	1667 (36.7)
Don’t know/don’t want to report	15 (0.3)	10 (0.3)	15 (0.3)	16 (0.4)
**Physical activity**				
Never/seldom	2517 (55.0)	2017 (53.2	2480 (55.0)	2497 (55.0)
Regular (≥ 2 h/week)	1989 (43.5)	1738 (45.9)	1969 (43.6)	1972 (43.5)
Don’t know/don’t want to report	68 (1.5)	33 (0.9)	64 (1.4)	69 (1.5)
**Educational status**				
No university degree	2222 (48.6)	1763 (46.5)	2183 (48.4)	2200 (48.5)
University degree	2342 (51.2)	2023 (53.4)	2320 (51.4)	2329 (51.3)
Don’t know/don’t want to report	10 (0.2)	2 (0.1)	10 (0.2)	9 (0.2)

All values reported as n (% group) unless otherwise specified.

PSQI, Pittsburgh Sleep Quality Index; n, number; DIS, difficulty initiating sleep; DSA, difficulty staying asleep; DSA; difficulty staying asleep; BMI, body mass index.

The population median sleep duration was 7 h/night, and 34.8% of the sleep duration subsample participants reported that they habitually slept less than 7 h per day. 50.4% reported low sleep quality (i.e., PSQI score > 5) in the PSQI cohort. In the subsample to investigate the association between trouble falling asleep and pet ownership/dog walking, 11.2% reported difficulty falling asleep. Finally, 38.9% of the middle-aged to older subjects complained about difficulty staying asleep.

### Association between pet ownership and sleep

The adjusted logistic regression analyses revealed no significant associations between pet ownership (any pet vs no pet) and sleep outcomes. Similar null-results were observed when comparing dog owners with non-dog owners. In contrast, we found that cat owners have a higher odds ratio of failing to achieve the recommended duration of 7 h of sleep per day (1.18 [1.02, 1.37] vs non-cat owners, *P* = 0.028). No cat ownership associations with sleep quality, difficulty falling asleep, and difficulty staying asleep were found. Please see Table 2.

**Table 2:** Association between pet ownership and sleep in the SCAPIS cohort (Uppsala).

	Pet owner	Non-pet owner	Cat owner	Non-cat owner	Dog owner	Non-dog owner
	OR [95% CI], *P*		OR [95% CI], *P*		OR [95% CI], *P*	
**Odds of experiencing poor sleep quality (PSQI > 5)**						
No. of subjects	1406	2382	863	2925	613	3175
PSQI > 5 (%)	51.7	49.6	53.3	49.5	50.6	50.4
Fully adjusted^a^	1.00 [0.87,1.15], * P* = 0.997	1	1.11 [0.94,1.30], * P* = 0.22	1	0.92 [0.76,1.10], * P* = 0.35	1
**Odds of having difficulty with initiating sleep at night**						
No. of subjects	1648	2865	1011	3502	728	3785
DIS (%)	11.4	11.1	12.5	10.8	11.1	11.2
Fully adjusted^a^	0.93 [0.76,1.14], * P* = 0.51	1	1.08 [0.86,1.35], * P* = 0.52	1	0.89 [0.68,1.16], * P* = 0.38	1
**Odds of having difficulty with staying asleep at night**						
No. of subjects	1657	2881	1017	3521	736	3802
DSA (%)	39.6	38.5	40.4	38.5	40.2	38.7
Fully adjusted^a^	1.01 [0.89,1.15], * P* = 0.84	1	1.05 [0.91,1.22], * P* = 0.49	1	1.01 [0.86,1.20], * P* = 0.89	1
**Odds of sleeping < 7 h per day**						
No. of subjects	1669	2905	1026	3548	741	3833
< 7 h/day (%)	36.1	34.0	38.6	33.7	35.4	34.7
Fully adjusted^a^	1.04 [0.91,1.18], * P* = 0.60	1	**1.18 [1.02,1.37], P = 0.028**	1	0.96 [0.81,1.13], * P* = 0.59	1

CI, confidence interval; DIS, difficulty initiating sleep; DSA, difficulty staying asleep; No., number; OR, odds ratio; PSQI, Pittsburgh Sleep Quality Index.

^a^Logistic regression was adjusted for the following variables: sex, education, age, BMI, social interaction, regular physical activity, alcohol frequency, socio-economic stability, night shift work, diabetes, sleep apnea, constant stress, current smoking.

Another aim of the present study was to investigate the association of dog walking engagement with sleep. When splitting dog owners into subgroups based on dog walking engagement, no significant associations with the sleep outcomes emerged (Table 3).

**Table 3:** Logistic regression analysis to investigate the association between dog walking and sleep in the SCAPIS cohort (Uppsala).

	Majority of dog walking	About half of dog walking	Lesser part of dog walking	Non-dog owners
	OR [95% CI], *P*	OR [95% CI], *P*	OR [95% CI], *P*	
**Odds of experiencing poor sleep quality (i.e., PSQI > 5)**				
No. of subjects	217	198	182	3175
PSQI > 5 (% group)	49.3	52.5	48.4	50.4
Fully adjusted^a^	0.81 [0.61,1.09], * P* = 0.17	1.06 [0.78,1.43], * P* = 0.71	0.86 [0.63,1.18], * P* = 0.34	1
**Odds of having difficulty with initiating sleep at night**				
No. of subjects	261	228	217	3785
DIS (% group)	12.3	10.5	10.6	11.2
Fully adjusted^a^	0.93 [0.62,1.40], * P* = 0.74	0.90 [0.57,1.42], * P* = 0.74	0.86 [0.54,1.37], * P* = 0.53	1
**Odds of having difficulty with staying asleep at night**				
No. of subjects	265	228	221	3802
DSA (% group)	45.7	40.8	33.5	38.7
Fully adjusted^a^	1.18 [0.92,1.53], * P* = 0.20	1.09 [0.83,1.45], * P* = 0.53	0.79[ 0.58,1.06], * P* = 0.11	1
**Odds of sleeping < 7 h per day**				
No. of subjects	265	229	224	3833
< 7 h/d (% group)	35.1	33.2	36.2	34.7
Fully adjusted^a^	0.94 [0.72,1.23], * P* = 0.65	0.90 [0.68,1.20], * P* = 0.47	0.97 [0.73,1.29], * P* = 0.82	1

CI, confidence interval; DIS, difficulty initiating sleep; DSA, difficulty staying asleep; No., number; OR, odds ratio; PSQI, Pittsburgh Sleep Quality Index.

^a^Logistic regression was adjusted for the following variables: sex, education, age, BMI, social interaction, regular physical activity, alcohol frequency, socio-economic stability, night shift work, diabetes, sleep apnea, constant stress, current smoking.

Overall, no significant interactions between pet ownership and sex were found for any of the sleep outcomes (pet ownership*sex: *P* ≥ 0.14; dog ownership*sex: *P* ≥ 0.42; cat ownership*sex: *P* ≥ 0.46; and dog walking*sex: *P* ≥ 0.23). Note that similar null-results for the association between the exposure and outcome variables were found when using unadjusted logistic regression analyses, except for a significant association between dog walking and difficulty staying asleep (1.33 [1.04,1.71] compared to non-dog owners, *P* = 0.025).

## Discussion

In the present study, we found a significant association between cat ownership and not reaching 7 h of sleep per night, as recommended for adults. In contrast, owning a dog, the second most prevalent pet in our cohort, was not associated with sleep. We neither found an association between general pet ownership and sleep. These findings might suggest that certain pet groups have a greater impact on the owner’s sleep than others.

Our results did not show any association between general pet ownership and sleep. They contrast with previous findings from the Whitehall II study, where pet ownership was associated with less trouble falling asleep but less refreshed awakening^1^. Possible explanations for these contrasting results could relate to differences in sample size, age range, how subjective sleep was measured, and adjustments of confounders.

Our findings do not suggest that general pet ownership alters sleep. However, it must be kept in mind that pets may, under certain circumstances, still improve the owner’s sleep, e.g., among people suffering from anxiety, depression, loneliness, and grief. For instance, in a study involving 340 older adults from China, it was demonstrated that pet engagement eased depression^11^, which can cause sleep problems^12^.

It is also possible that pet ownership can impair sleep. For instance, in a previous study, one-fifth of the pet owners reported that their co-sleeping pet disrupted their sleep^4^. Extending these findings, we found that cat owners exhibited an increased risk of sleeping too short. Cats can display crepuscular behaviour, i.e., they are primarily active at dawn and dusk. Consequently, when co-sleeping with a cat, the owner’s sleep might become disrupted.

Considering that owning a dog might be particularly beneficial to sleep, e.g., due to increased physical activity and spending time outside, we also investigated if there is a link between dog ownership and dog walking engagement with sleep. Contrary to our expectations, we found no associations when controlling for confounders known to affect sleep, such as chronic stress, shift work, and lack of social interaction. We did not have information about the breed, age, and pedigree of dogs, which may moderate the association between dog ownership and health outcomes, including sleep. For instance, owning a dog of mixed pedigree or a dog belonging to the ‘companion/toy’ breed group was associated with hypertension and dyslipidemia in the dog owner^13^. Additionally, owning a dog from the ‘Spitz/primitive’ breed and the combined group of’ active dog breeds’ was associated with a lower risk of diabetes mellitus^13^. Altogether, our study does not provide compelling evidence for the hypothesis that dog walking may result in overall better sleep.

Several strengths and limitations apply to our study. The primary strength of the present study is its large sample size. Moreover, results were robust to adjustments for multiple potential confounders, such as participants’ age and educational level, yet a possibility of residual confounding remains. Another strength is that the PSQI^5^, a widely used questionnaire to assess a person’s sleep quality, was used in the present analysis. On the other hand, the accuracy of self-reported sleep length might be subject to recall bias. Another limitation of the current study is the restricted age range (50–65), limiting the generalisability of the results. Besides, we have no information regarding pet engagement (except dog walking). For instance, co-sleeping with pets can be perceived as disruptive to sleep^4^. In SCAPIS, it was not surveyed either whether non-pet owners may have regular contact with visiting pets. A study involving 100 residents from nursing homes (ages ranged between 79 and 90 years) showed that their sleep duration was increased when a dog accompanied visitors^14^. When interpreting our results, it must be borne in mind that the observed association between cat ownership and short sleep duration might be a chance finding. Thus, this observation should be seen as hypothesis-generating only. Finally, although our findings show an association between cat ownership and short sleep duration, these observational results do not imply a causal relationship.

## Conclusions

We found that owning a cat, the most prevalent pet group in the present study, was associated with increased odds of sleeping less than the recommended seven hours per day. Whether this means that cats represent a risk factor for short sleep duration cannot be derived from the present observational study. Therefore, future studies should more thoroughly investigate the various aspects of cat ownership, e.g., the cat’s breed, age, and co-sleeping with the cat.

## Supplementary Information

Supplementary Figure S1.

## References

[1] Mein G, Grant R (2018). A cross-sectional exploratory analysis between pet ownership, sleep, exercise, health and neighbourhood perceptions: The Whitehall II cohort study. BMC Geriatr..

[2] Defelipe R, Savalli C, Otta E (2020). Demographics and self-reported well-being of Brazilian adults as a function of pet ownership: A pilot study. Heliyon.

[3] Patel SI (2017). The effect of dogs on human sleep in the home sleep environment. Mayo Clin. Proc..

[4] Krahn LE, Tovar MD, Miller B (2015). Are pets in the bedroom a problem?. Mayo Clin. Proc..

[5] Buysse DJ, Reynolds CF, Monk TH, Berman SR, Kupfer DJ (1989). The Pittsburgh sleep quality index: A new instrument for psychiatric practice and research. Psychiatry Res..

[6] Tan X, van Egmond LT, Cedernaes J, Benedict C (2020). The role of exercise-induced peripheral factors in sleep regulation. Mol. Metab..

[7] Jurado-Fasoli L (2020). Exercise training improves sleep quality: A randomized controlled trial. Eur. J. Clin. Invest..

[8] Tan X, Alén M, Wiklund P, Partinen M, Cheng S (2016). Effects of aerobic exercise on home-based sleep among overweight and obese men with chronic insomnia symptoms: a randomized controlled trial. Sleep Med..

[9] Bergström G (2015). The Swedish CArdioPulmonary BioImage Study: Objectives and design. J. Intern. Med..

[10] Textor J, Hardt J, Knüppel S (2011). DAGitty: A graphical tool for analyzing causal diagrams. Epidemiology.

[11] Cheung C, Kam PK (2018). Conditions for pets to prevent depression in older adults. Aging Ment. Heal..

[12] Pandi-Perumal SR (2020). Clarifying the role of sleep in depression: A narrative review. Psychiatry Res..

[13] Mubanga M (2019). Dog ownership and cardiovascular risk factors: a nationwide prospective register-based cohort study. BMJ Open.

[14] Thodberg K (2016). Therapeutic effects of dog visits in nursing homes for the elderly. Psychogeriatrics.

