# Supplementary material for: Association between pet ownership and sleep in the Swedish CArdioPulmonary bioImage Study (SCAPIS)
Source: Sci Rep. 2021 Apr 2;11:7468. doi: 10.1038/s41598-021-87080-7 (PMC8018946; doi:10.1038/s41598-021-87080-7)
Supplement: Supplementary file 1 — Supplementary Figure S1. [file 41598_2021_87080_MOESM1_ESM.docx]

**Association between pet ownership and sleep in the Swedish CArdioPulmonary bioImage Study (SCAPIS) - Supplement**

Lieve T. van Egmond, MSc^1*^, Olga E. Titova, PhD^2^, Eva Lindberg, MD, PhD^3^,

Tove Fall, PhD^4^, Christian Benedict, PhD^1^

^1^Department of Neuroscience, Uppsala University, Uppsala, Sweden

^2^Department of Surgical Sciences, Unit of Medical Epidemiology, Uppsala University, Uppsala, Sweden

^3^Department of Medical Sciences, Respiratory, Allergy and Sleep Research, Uppsala University, Uppsala, Sweden

^4^Department of Medical Sciences, Molecular Epidemiology and Science for Life Laboratory, Uppsala University, Uppsala, Sweden

* To whom correspondence should be addressed:

Department of Neuroscience, Uppsala University

Husargatan 3, Box 593, 751 24 Uppsala, Sweden

Phone: 0046-18-471-4326

[lieve.van.egmond@neuro.uu.se](mailto:lieve.van.egmond@neuro.uu.se)

**Supplemental Figure 1: Directed acyclic graph of the pet-ownership – sleep relationship.** Green paths show causal pathways, pink paths show biasing paths.

**
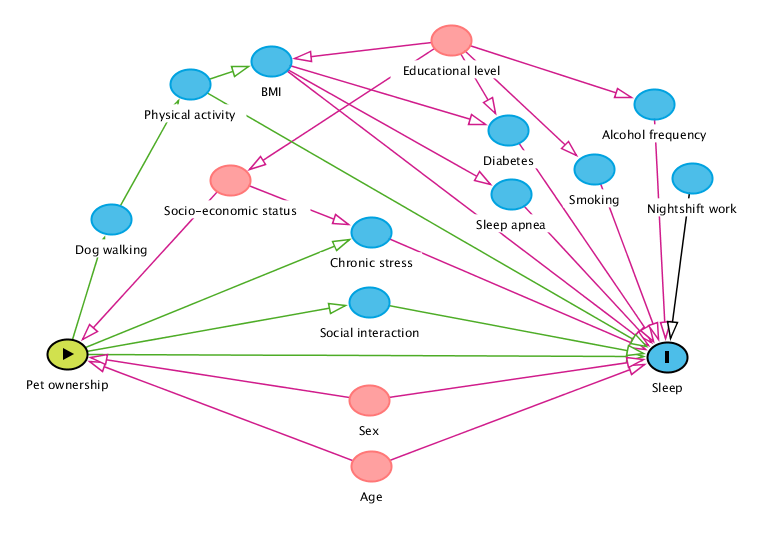
**
